# 左肺恶性外周神经鞘瘤1例

**DOI:** 10.3779/j.issn.1009-3419.2012.07.10

**Published:** 2012-07-20

**Authors:** 剑 褚, 秀 陈, 东涛 尹, 道喜 王, 冰 韩, 娜 刘

**Affiliations:** 100088 北京，第二炮兵总医院心胸外科 Department of Cardiothoracic Surgery, the Second Artillery General Hospital of PLA, Beijing 100088, China

恶性外周神经鞘瘤（malignant peripheral nerve sheath tumor, MPNST）是一种来源于周围神经的恶性肿瘤，全身多处器官均可生长，恶性程度高，预后差，手术完整切除肿瘤是唯一治疗方法。北京第二炮兵总医院心胸外科收治1例左下肺原发MPNST患者，经手术完整切除，实为罕见，现报告如下。

## 1 临床资料

患者，女，58岁。因胸闷、咳嗽、咳痰、痰中带血1个月入院。查体气管右移，左下肺叩诊浊音，听诊呼吸音减弱。胸部增强CT（图 1）示左下肺有一大小约13 cm×10 cm×8 cm占位，边界光整，病灶中实性部分呈轻度强化征象。左侧甲状腺弥漫性增大，内可见团片状低密度与斑点状高密度影，气管受压右移。胸部MRI示左侧胸腔内一巨大不规则混杂信号病变，边界清晰，大小约为13 cm×10 cm×8 cm，病变未与同水平椎体相连。左侧胸腔内可见长T2液体信号。头颅MRI提示双侧基底节区腔隙性脑梗塞；脑白质脱髓鞘（轻度）；副鼻窦炎。胸椎MRI示胸椎退行性改变。腹部超声示脂肪肝。肺功能提示：通气功能正常，最大自主通气量轻度下降，通气储量百分比中度不足；弥散功能轻度下降，残总比轻度增高；总气道和中心气道粘性阻力均轻度增高。动脉血气分析示：PH 7.437，PCO_2_ 42.7 mmHg，PO_2_ 68 mmHg，HCO_3_ 28.8 mmol/L，BEecf 5 mmol/L，SO_2_ 94%。完善术前检查后于2011年12月在全麻下行左肺下叶切除加纵隔淋巴结清扫术。由于患者气管受压狭窄，考虑行双腔气管导管插管困难，遂行单腔气管导管插管，患者右侧卧位，左后外侧切口，长约18 cm，第六肋床入胸。见胸内少量积液，淡黄色，约20 mL，左肺下叶内可及一约13 cm×12 cm×10 cm大小肿块，下叶几乎被整个瘤体占据，质中硬，似有囊性感，境界不清，但肺叶表面脏层胸膜无明显肿瘤外侵，带有瘤体的左肺下叶占据整个左下胸腔，无游离度，与周围毗邻胸壁壁层胸膜仅有少量索条状粘连。主动脉弓旁淋巴结肿大融合，直径约1.0 cm-2.0 cm，质中硬，活动。由于切口相对不大，瘤体巨大，操作空间小，无法接近肺门，由麻醉师在纤维支气管镜引导下将气管导管向下插入右主支气管内改为单肺通气。于腋中线第八肋间置入胸腔镜观察瘤体与周围关系，并协助分离下肺韧带。分开下叶前后之不全肺裂，于斜裂内找出下肺动脉之背段支及基底段支两支，分别予以结扎切断。因无法显露下肺静脉，首先分出左肺下叶支气管，用切割器切断，最后将下肺静脉用切割器切断，移除左肺下叶（图 2）。术中冰冻病理示恶性肿瘤，遂行纵隔淋巴结清扫后完成手术，术中出血约200 mL，胸腔内胸壁与肿块毗邻面无明显渗出。术后病理巨检见左下肺灰红灰褐色类圆形肿物1枚，体积13 cm×12 cm×10 cm，切面灰白灰红色质软实性，多为坏死组织。病理诊断为MPNST（图 3），淋巴结未见转移。免疫组化示GFAP（-），NSE（-），Syn（+），SMA（-），Vim（+），CK（-），CK5/6（-），CK8/18（-），TTF-1（-），CD34（-），S-100（+），CD10（-），CD31（-），CD99（-），CD117（-），CR（-），MC（-），Ki67（-）（50%）。术后恢复良好，术后第1日胸腔引流液为150 mL。术后未行其它辅助治疗，出院后随访3个月，情况良好，无胸部不适，复查胸部CT（图 4）未见异常。

**1:**
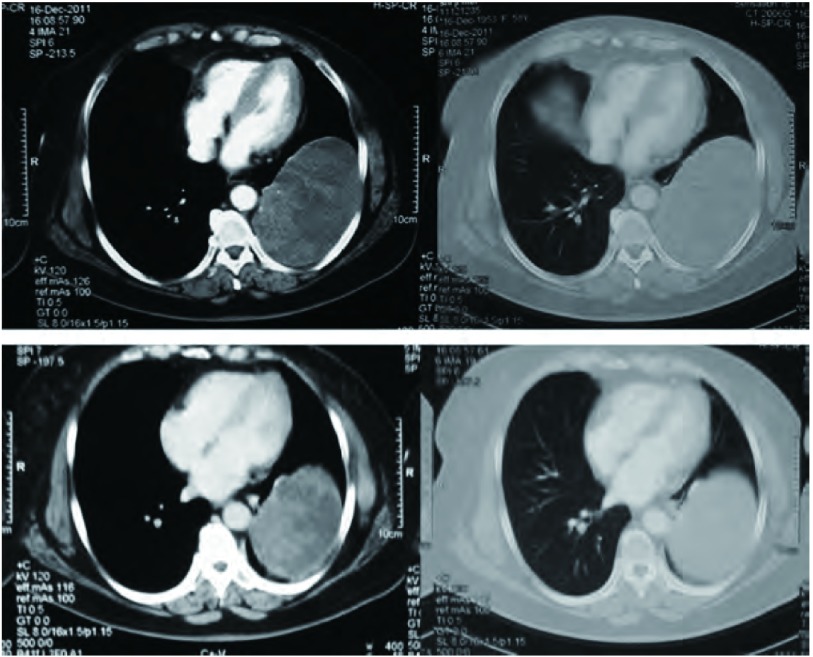
胸部CT增强示左下肺有一大小约13 cm×10 cm×8 cm的占位，边界光整，病灶中实性部分呈轻度强化征象。 Contrast enhanced CT scan of the chest: a large mass (13 cm×10 cm×8 cm) in the left lower lobe, with clear boundaries and smooth fringes. The solid portion of the mass shows a slight contrast enhancement.

**2:**
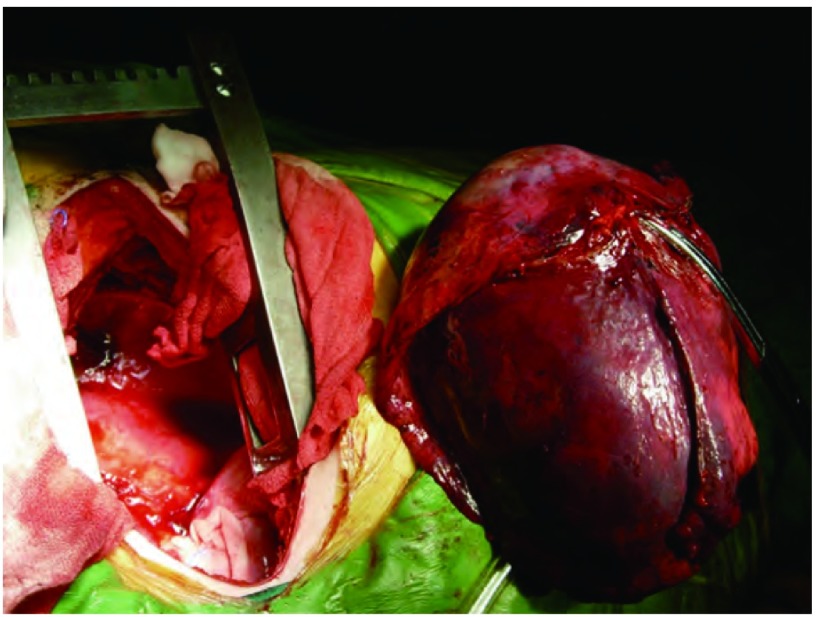
手术切除左肺下叶 Left lower lobectomy

**3:**
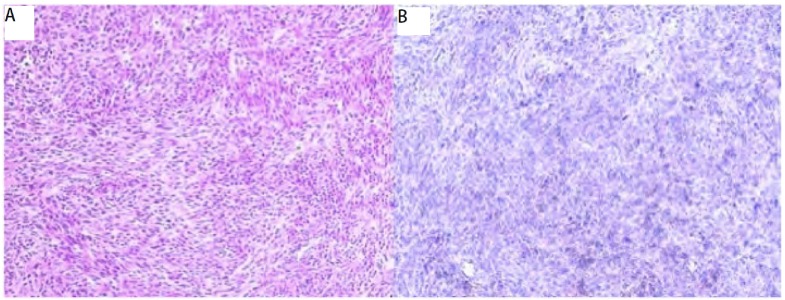
病理诊断：恶性外周神经鞘瘤。A：光镜下显示小的梭型细胞，呈密集栅栏状排列（HE, ×400）；B：免疫组织化学检查示S-100蛋白表达阳性（EnVision, ×400）。 Pathologic diagnosis: malignant peripheral nerve sheath tumor. A: Microphotograph is showing spindle cell tumor arranged in a dense palisading pattern (HE, ×400); B: Immunohistochemistry shows positivity of tumor cells to S-100 (EnVision, ×400).

**4:**
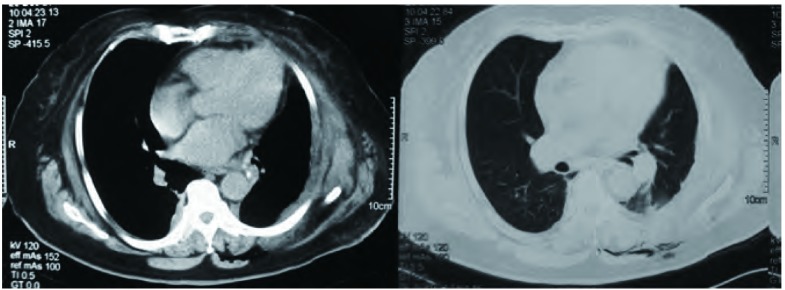
术后复查胸部CT示肿瘤被完全切除 CT scan of the chest showed that the tumor was completely removed after operation

## 2 讨论

MPNST是一种极为罕见的肺内恶性肿瘤。文献^[1]^报道MPNST多见于成年人，无性别差异，常见症状有胸痛、呼吸困难、咳嗽、咯血。影像学检查如X线、CT和MRI均可发现肺内肿块，但不具有特异性，临床上诊断较为困难。细针穿刺活检结果不确切，需手术切除进行组织学检查才能确诊。此瘤的组织学形态要与原发肺的恶性纤维组织细胞瘤、平滑肌肉瘤和纤维肉瘤鉴别，常规苏木素-伊红（hematoxylin-eosin, HE）切片常难鉴别，免疫组织化学是重要的辅助诊断和鉴别诊断的手段，S-100蛋白为相对特异性标志物，大约50%-70%的MPNST呈局灶性阳性反应^[2]^。本病生物学特征为易局部侵犯和血行转移，治疗原则是完整手术切除及长期随访，切除不彻底者易原位复发。预后与肿瘤部位的深浅、病理分化高低及侵犯范围、手术切除情况有关，与术后放化疗的关系尚需进一步探讨。

胸内肿瘤直径 > 10 cm者被称为胸内巨大肿瘤，瘤体巨大者常与周围血管及脏器关系密切，手术切除难度较大，术中应注意：①首选双腔气管导管插管，如气管受压狭窄插管困难者，可在纤维支气管镜引导下将单腔气管导管插入一侧主支气管，从而实现单肺通气。也有报道^[3]^在体外循环辅助下顺利实施手术；②控制出血，尽量完整切除，避免分次切除，准备好足够静脉通路和血源，文献^[4]^报道在肿瘤切除术前行滋养血管介入栓塞治疗可明显减少术中出血量，并有效缩短手术时间；③合理选择切口，由于肿瘤巨大，常无间隙进行操作致显露、解剖、切除均较困难，以往多采用延长切口或多个切口分次逐个切除^[5, 6]^，手术操作困难，费时且创伤大，容易导致肿瘤复发和转移。本文作者推荐采用电视胸腔镜（video-assisted thoracoscopic surgery, VATS）辅助下胸部小切口切除肺部巨大肿瘤，方便术中探查了解肿瘤与周边情况，有利于肿瘤的游离及完整切除，创伤小且术后恢复快^[7]^。
